# A Study on the Etiology and Clinical Manifestations of Community-Acquired Pneumonia in Adults in Western India

**DOI:** 10.7759/cureus.63132

**Published:** 2024-06-25

**Authors:** Vikram B Vikhe, Ahsan A Faruqi, Rahul S Patil, Harshad Patel, Devansh Khandol, Avani Reddy

**Affiliations:** 1 General Medicine, Dr. D. Y. Patil Medical College, Hospital and Research Centre, Dr. D. Y. Patil Vidyapeeth (Deemed to be University), Pune, IND

**Keywords:** sputum culture, prospective cohort, pune, clinical manifestations, adult pneumonia, western india, streptococcus pneumoniae, pneumonia, klebsiella pneumoniae (kp), community-acquired pneumonia (cap)

## Abstract

Background: Community-acquired pneumonia (CAP) is an acute lung infection affecting the alveoli in individuals who have not had recent exposure to healthcare settings. It is characterized by newly detected pulmonary infiltration on a chest X-ray or computed tomography scan, accompanied by at least two of the following symptoms: a new or worsening cough, shortness of breath, increased sputum production, fever or hypothermia, pleuritic chest pain, hypoxia, confusion, or an abnormal WBC count (either leukopenia or leukocytosis). It is a major contributor to global mortality and morbidity, especially in elderly populations. This study aims to investigate the etiology of CAP in our region and analyze the clinical characteristics of patients diagnosed with CAP.

Methodology: This prospective, hospital-based study was conducted at Dr. D. Y. Patil Medical College, Hospital and Research Centre, Pune, a 2,011-bed multispecialty hospital. The study included 100 patients over 18 years old, diagnosed with CAP, and hospitalized between January 2023 and January 2024. All patients underwent a thorough clinical assessment, and sputum cultures were collected on the day of admission. Patients under 18 years old, those who had been hospitalized within the preceding two weeks, individuals with pneumonia caused by tuberculosis or aspiration pneumonia, patients with compromised immune systems, and pregnant women were excluded.

Results: The study included 100 patients with a mean age of 53.13 years (±18.31). The most common age group was 59-68 years, which included 25 (25%) cases, followed by the 69-78 year age group with 18 (18%) cases and the 18-28 year age group with 15 (15%) cases. The majority were male, with 61 (61%) cases. Common symptoms included fever in 78 cases (78%), chest pain in 69 cases (69%), dyspnea in 65 cases (65%), and cough in 51 cases (51%). Sputum cultures showed growth in 65 cases (65%), with *Klebsiella pneumoniae* being the most prevalent pathogen in 28 cases (43%), followed by *Streptococcus pneumoniae* in 18 cases (28%). Together, these two pathogens accounted for 46 out of 65 positive samples (70%).

Conclusions: This study highlights the clinical profile and rising etiology of* K. pneumoniae* in CAP in adults in Western India, particularly in the elderly. These findings underscore the need for periodic updates on CAP etiology to inform empirical treatment strategies effectively. Future research should use advanced diagnostics and diverse samples to refine CAP management, with continuous monitoring to update treatment protocols.

## Introduction

Community-acquired pneumonia (CAP) is an acute lung infection that affects the alveoli in individuals without recent exposure to healthcare settings [1]. Pneumonia is often described in medical literature as a newly detected pulmonary infiltration on a chest X-ray or computed tomography (CT) scan, accompanied by at least two of the following symptoms: a new or worsening cough, shortness of breath, increased sputum production, fever, or hypothermia, pleuritic chest pain, hypoxia, confusion, or an abnormal WBC count (either leukopenia or leukocytosis) [1-4]. CAP is a significant contributor to global mortality and morbidity and ranks among the leading causes of death from infectious diseases. In the United States, the occurrence of CAP among adults under 65 years old varies between 2.48 and 10.6 cases per thousand person-years [4,5]. As expected, the incidence is higher among the elderly, with rates of 6.3 cases per thousand person-years in those aged 65-79, increasing to 16.43 cases per thousand person-years in individuals over 80 [4,5]. India bears 23% of the world's pneumonia cases, with fatality rates varying from 14% to 30% [6].

*Streptococcus pneumoniae* remains a leading cause of CAP globally across all age groups. Other significant pathogens include *Staphylococcus aureus*, *Pseudomonas aeruginosa*, *Haemophilus influenzae*, and atypical bacteria such as *Chlamydia pneumoniae*, *Mycoplasma* *pneumoniae*, and *Legionella pneumophila *[6,7]. The bacterial causes of CAP vary with geographic location and patient characteristics, influenced by factors like laboratory test utilization, healthcare access, testing guidelines, and available laboratory facilities. In India, *S. pneumoniae* is the most frequent cause of CAP [6,7]. Other significant pathogens include *M. pneumoniae*, *Klebsiella pneumoniae*, and *L. pneumophila* [7]. Notably, *K. pneumoniae*, previously uncommon in CAP cases in India, is now emerging as a common pathogen [6-9].

Common symptoms of CAP include fatigue, dyspnea, cough, chills, fever (core body temperature of 38°C), rigors, pleuritic chest pain, and new focal chest signs without another obvious cause. In certain subgroups, such as the elderly, the clinical presentation may lack these classic symptoms and instead involve an altered state of consciousness or gastrointestinal discomfort, often without fever, which can delay diagnosis [1,6,10,11].

This study aimed to investigate the etiology of CAP in our region and analyze the clinical characteristics of patients diagnosed with CAP.

## Materials and methods

Study design and setting

**Figure 1:**
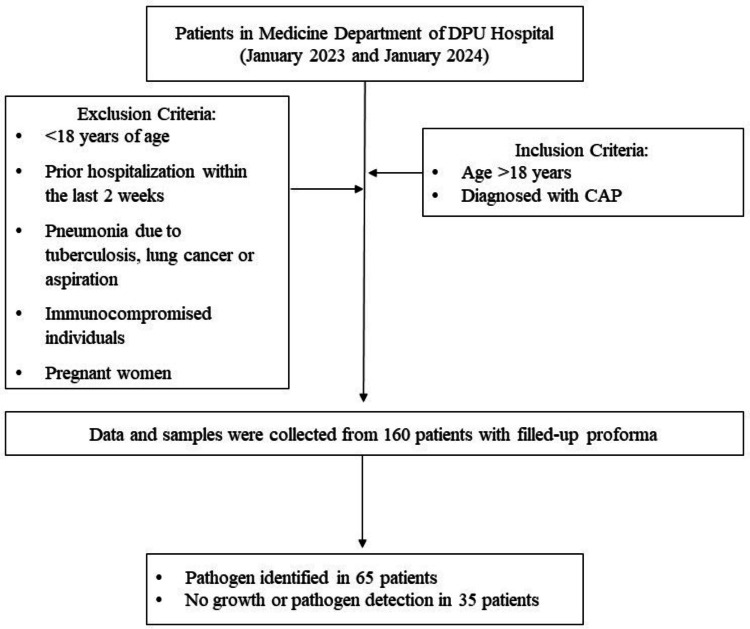
Flow diagram of the study DPU: Dr. D. Y. Patil University; CAP: community-acquired pneumonia

This prospective observational cohort, hospital-based study was conducted at Dr. D. Y. Patil Medical College, Hospital and Research Centre, Pune, a 2,011-bed multispecialty hospital. The study included 100 patients aged over 18 years, with a primary diagnosis of CAP, who were hospitalized between January 2023 and January 2024 (Figure 1). Each participant underwent a thorough clinical assessment and investigation. The study received approval from the Institutional Ethics Subcommittee of Dr. D. Y. Patil Medical College, Hospital and Research Centre (approval number IESC/PGS/2022/03, dated September 28, 2022).

Inclusion criteria

The inclusion criteria included patients aged over 18 years, diagnosed with CAP, attending the medical outpatient department, and admitted with a clinical diagnosis of CAP.

Exclusion criteria

The exclusion criteria included patients under 18 years, those hospitalized within the preceding two weeks, individuals with pneumonia caused by tuberculosis or aspiration, individuals with compromised immune systems, and pregnant women.

Sample size

Based on the prevalence of *K. pneumoniae* (22%) reported in the study by Bansal et al. [12] and considering a 95% confidence interval with an acceptable difference of 8%, a sample size of 100 was calculated. This calculation was performed using the WinPepi software, version 11.38 (J. H. Abramson, Brixton Health, United Kingdom).

Data and sample collection

A detailed clinical history was taken from all patients using a pretested pro forma, focusing on symptoms of CAP. Patients underwent physical examinations and reviewed their available laboratory reports, with relevant findings such as X-ray and biochemistry results noted. Blood investigations included a complete blood picture, serum electrolytes, renal function tests, blood glucose levels, and other relevant tests to exclude other differentials. Radiological diagnosis involved chest X-rays and, if necessary, CT scans. Patients were diagnosed according to the Infectious Diseases Society of America/American Thoracic Society consensus guidelines [11]. Sputum samples were collected in a clean and sterile container after demonstrating the proper method for expectorating, ensuring collection before antibiotic administration. For patients unable to expectorate spontaneously, nebulization with a 3% hypertonic saline solution was used to induce sputum production. These samples were inoculated in three agar media: Columbia blood agar, MacConkey agar, and chocolate agar, as described in standard guidelines [13]. All culture media and discs were made by Becton Dickinson, Sparks, MD. Additionally, sputum was tested for acid-fast bacilli and GeneXpert (Cepheid, Sunnyvale, CA) in all patients to exclude tuberculosis.

Consent

Ethical approval and informed consent procedures were stringently followed to ensure institutional and international ethical standards adherence. Participants were fully informed about the study's aims, procedures, potential risks, and benefits in their native language, and written consent was obtained before inclusion. This rigorous process ensured ethical integrity and participant autonomy throughout the study.

Statistical analysis

Categorical variables were presented as frequencies and percentages. As this is a descriptive study, tests of statistical significance and hypothesis testing were not considered. Data tracking was performed using Microsoft Excel (Microsoft Corporation, Redmond, WA) and analyzed using IBM SPSS Statistics version 29.0.2.0 (IBM Corp., Armonk, NY).

## Results

Our study included 100 patients. Table 1 summarizes their comorbidities, risk factors, clinical manifestations, and basic demographics.

**Table 1:** Baseline data N: total number of subjects, i.e., 100; IHD: ischemic heart disease; COPD: chronic obstructive pulmonary disease

Characteristics	N (%)
Sex	
Male	61 (61)
Female	39 (39)
Risk factors	
Smoker	41 (41)
Tobacco chewer	61 (61)
Alcohol user	44 (44)
>60 years age	48 (48)
Clinical manifestations	
Fever	78 (78)
Dyspnea	65 (65)
Cough	51 (51)
Expectoration	60 (60)
Hemoptysis	2 (2)
Chest pain	69 (69)
Comorbidities	
Diabetes	34 (34)
Hypertension	28 (28)
IHD	7 (7)
Liver disease	4 (4)
COPD	7 (7)

**Figure 2:**
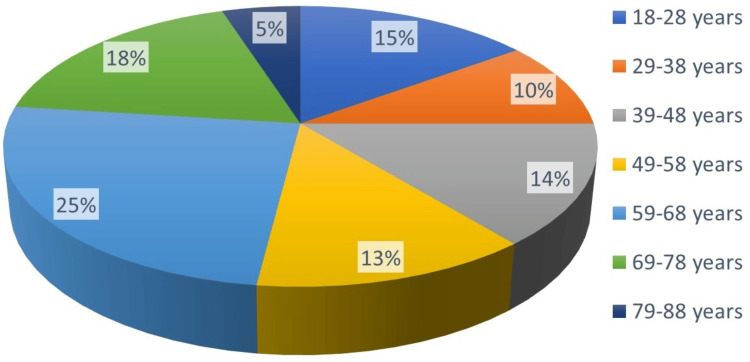
Pie chart showing the agewise distribution of patients in the study

The mean age of patients in our study was 53.13 years (±18.31). Males outnumbered females with a ratio of 1.56:1. Figure 2 illustrates that the most prevalent age group for CAP in this study was between 59 and 68 years, which included 25 patients (25%). This was followed by the age group of 69-78 years, which had 18 patients (18%), and the age group of 18-28 years, which had 15 patients (15%).

**Figure 3:**
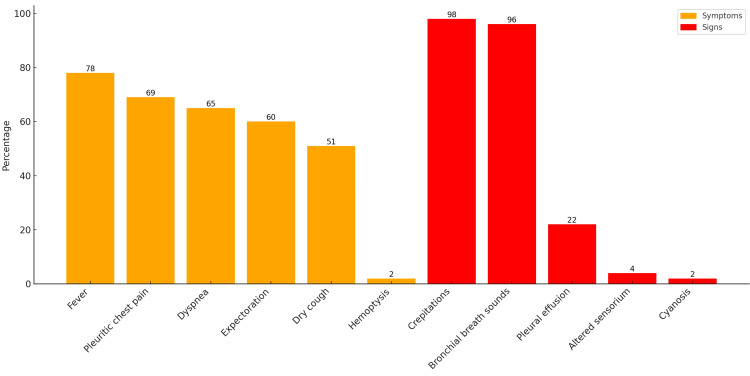
Clinical manifestations of CAP patients in the study CAP: community-acquired pneumonia

Figure 3 illustrates the prevalence of various symptoms and signs in patients diagnosed with CAP. Fever is the most prevalent symptom, affecting 78 cases (78%). This is followed by pleuritic chest pain and dyspnea, affecting 69 patients (69%) and 65 patients (65%), respectively. Other symptoms include expectoration, noted in 60 cases (60%), and dry cough, present in 51 cases (51%). Hemoptysis is rare and is observed in only two patients (2%).

Regarding clinical signs, crepitations are the most predominant, present in 98 cases (98%). Bronchial breath sounds are observed in 96 patients (96%). Pleural effusion is seen in 22 cases (22%). Altered sensorium is seen in four patients (4%), and cyanosis affects only two patients (2%). Overall, the data highlight that fever, pleuritic chest pain, and dyspnea are key symptoms of CAP, while crepitations and bronchial breath sounds are the most common clinical signs.

**Figure 4:**
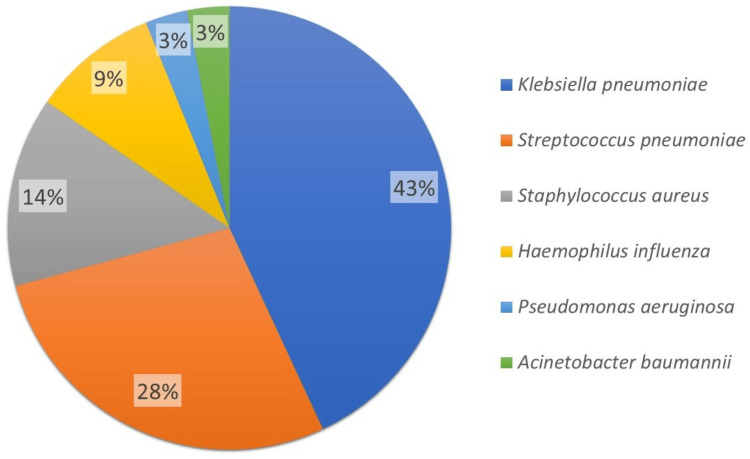
Pie chart showing the etiological pathogens isolated from the sputum cultures of CAP patients in this study CAP: community-acquired pneumonia

Figure 4 visualizes the distribution of organisms isolated from sputum samples from CAP patients in the study. Out of the total samples, 65 showed growth, indicating successful isolation of pathogens. *K. pneumoniae* was the most prevalent organism, identified in 28 cases (43%), followed by *S. pneumoniae* found in 18 cases (28%). *S. aureus* was identified in nine cases (14%), making it another notable contributor. *H. influenzae* was found in six cases (9%), while *P. aeruginosa* and *A. baumannii* were each found in two cases (3%). Notably, 35 samples showed no growth. This distribution highlights the predominance of *K. pneumoniae* and *S. pneumoniae* in these CAP patients, which together accounted for 46 out of 65 positive samples (70%).

## Discussion

Our research included 100 patients diagnosed with CAP at a tertiary-care hospital. The mean age of the patients was 53.13 years (±18.31), with 48 (48%) being over 60 years old. The cohort comprised 61 males (61%) and 39 females (39%). Males dominated the study group with a male-to-female ratio of 1.56:1. Lifestyle factors showed a high prevalence of tobacco chewers, 61 (61%), alcohol users, 44 (44%), and smokers, 41 (41%). Comorbid conditions were prevalent, with diabetes being present in 34 cases (34%), hypertension in 28 cases (28%), chronic obstructive pulmonary disease (COPD) in seven cases (7%), ischemic heart disease in seven cases (7%), and liver disease in four cases (4%). In a study conducted by Eshwarappa et al. in Southwest India, most participants were male, with an average age of 38.08 years [14]. The most frequently observed comorbidities included HIV, COPD, and a history of pulmonary tuberculosis [14]. This study highlights the regional differences in the age and comorbidities of CAP patients [14]. Similarly, Bansal et al.'s study in Shimla, Himachal Pradesh, reported a mean age of 52.77 years with a predominance of males and identified COPD as the most common comorbid condition. These findings are consistent with our observation of a significant male predominance and the presence of multiple comorbidities among CAP patients [12].

The most common symptoms in our study were fever in 78 cases (78%), followed by chest pain in 69 cases (69%), dyspnea in 65 cases (65%), expectoration in 60 cases (60%), and cough in 51 cases (51%), with hemoptysis being rare and observed in only two cases (2%). Clinical signs included crepitations in 98 cases (98%), bronchial breath sounds in 96 cases (96%), pleural effusion in 22 cases (22%), altered sensorium in four cases (4%), and cyanosis in two cases (2%). In a study by Eshwarappa et al., the most common symptom was fever, observed in 100 cases (100%), cough in 94 cases (94%), and expectoration in 63 cases (63%) [14]. Similarly, Bansal et al. found that the most common symptoms were cough in 68 (97.1%) out of 70 cases, expectoration in 61 cases (87.1%), and fever in 63 cases (90%) [12]. These comparisons underline the variability in symptom presentation across different studies and regions.

In our study, *K. pneumoniae* was the most prevalent organism, found in 28 cases (43%), followed by *S. pneumoniae* in 18 cases (28%). Together, *K. pneumoniae* and *S. pneumoniae* accounted for 46 out of 65 positive samples (70%). *S. aureus* was identified in nine cases (14%), followed by *H. influenzae* in six cases (9%), and *P. aeruginosa* and *A. baumannii*, each detected in two cases (3%). Notably, 35 samples (35%) showed no growth. Other studies have also observed a low diagnostic yield due to the limited use of advanced diagnostic tools. For example, research by Dorairaj et al. in Chennai and Jain et al. in Madhya Pradesh primarily relied on sputum and blood cultures, resulting in a lower diagnostic yield [15,16]. Mythri and Nataraju also attributed their low isolation rate to factors such as the unavailability of sputum samples and the prior use of antibiotics [8].

There has been a concerning rise in the prevalence of *K. pneumoniae* as the causative organism in CAP among Indian adults. This trend is consistent with findings from other studies, such as the one conducted by Mythri and Nataraju in Bengaluru, India, involving 72 patients [8] and by Kalita et al. in Northeast Asia, involving 574 subjects. In our study, *K. pneumoniae* was followed by *S. pneumoniae* [9]. Studies by Bansal et al. in Northern India (70 patients), Dagaonkar et al. in Mumbai (100 patients), Para et al. in Kashmir, Northern India (225 patients), and Nagesh Kumar et al. (122 patients) also identified *S. pneumoniae* as the most common pathogen [12,17-19]. Systematic reviews of Indian studies done by Eshwara et al. and Ghia et al. further highlighted *S. pneumoniae* as a common pathogen. In these reviews, the second most common organism was *K. pneumoniae*, followed by *S. aureus* and *P. aeruginosa*, which aligns with our study findings [6,7]. Notably, Mycoplasma and Chlamydia were also reported as common pathogens in their reviews [6,7], but these were not detected in our study due to the absence of serological testing for these organisms.

Limitations

Conducting the study at a single center limits the diversity of the sample, potentially missing regional variations. Only sputum cultures were taken, likely contributing to the low yield of pathogen identification. Utilizing blood cultures, bronchoalveolar lavage cultures, and serology testing for atypical pathogens could have provided a better yield. Furthermore, the lack of longitudinal follow-up data prevented the assessment of the impact of the identified pathogens on patient outcomes, such as recovery times, recurrence rates, and long-term health effects. Another significant limitation was the descriptive nature of the study, which did not include a control group, precluding the possibility of performing statistical analyses, such as calculating p values, to compare different groups and determine the statistical significance of the findings.

## Conclusions

This study provides an overview of the clinical profile and etiology of CAP in adult patients in Western India. Pneumonia is particularly common in the elderly, who require careful attention when presenting with symptoms such as fever and dyspnea. Physicians should always be vigilant for pneumonia in these patients. Our findings highlight a rising trend of *Klebsiella pneumoniae* as a significant pathogen in CAP cases. Therefore, empirical treatment should consider targeting this pathogen to improve patient outcomes. Future studies should focus on incorporating advanced diagnostic tools and expanding the sample diversity to further refine CAP management strategies. Continuous monitoring and updated research are vital for adapting treatment protocols and enhancing the quality of care for CAP patients.
